# Merkel Cell Polyomavirus and Merkel Cell Carcinoma

**DOI:** 10.3390/cancers12071774

**Published:** 2020-07-03

**Authors:** Valeria Pietropaolo, Carla Prezioso, Ugo Moens

**Affiliations:** 1 Department of Public Health and Infectious Diseases, “Sapienza” University, 00185 Rome, Italy; valeria.pietropaolo@uniroma1.it (V.P.); carla.prezioso@uniroma1.it (C.P.); 2 IRCSS San Raffaele Pisana, Microbiology of Chronic Neuro-Degenerative Pathologies, 00166 Rome, Italy; 3 Molecular Inflammation Research Group, Department of Medical Biology, Faculty of Health Sciences, University of Tromsø—The Arctic University of Norway, 9037 Tromsø, Norway

**Keywords:** biomarkers, cell tropism, signaling pathways, therapy, transgenic mice, tumorigenesis

## Abstract

Viruses are the cause of approximately 15% of all human cancers. Both RNA and DNA human tumor viruses have been identified, with Merkel cell polyomavirus being the most recent one to be linked to cancer. This virus is associated with about 80% of Merkel cell carcinomas, a rare, but aggressive cutaneous malignancy. Despite its name, the cells of origin of this tumor may not be Merkel cells. This review provides an update on the structure and life cycle, cell tropism and epidemiology of the virus and its oncogenic properties. Putative strategies to prevent viral infection or treat virus-positive Merkel cell carcinoma patients are discussed.

## 1. Introduction

### 1.1. Genome MCPyV

**Figure 1:**
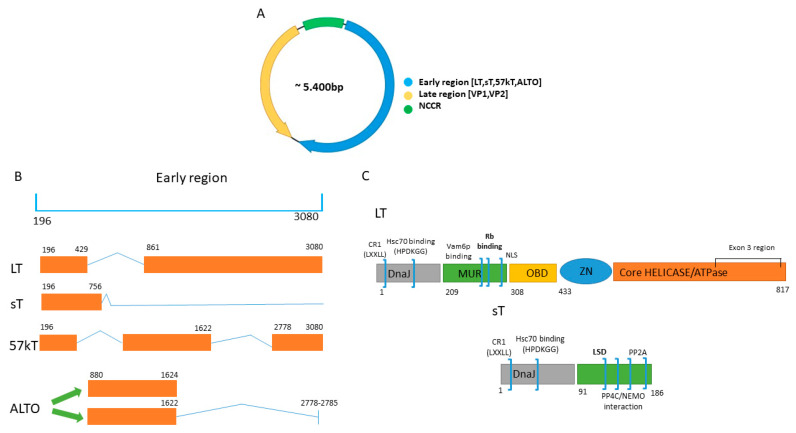
Structure of the MCPyV genome and the early region transcripts and the early proteins large T antigen (LT) and small T antigen (sT) with their functional domains. (**A**) Schematic presentation of the ~5400 bp circular dsDNA genome that includes a non-coding region (NCCR), an early region encoding T antigens that coordinate viral replication, and a late region containing the genes for the viral capsid proteins VP1 and VP2. (**B**) Multiple transcripts are generated from the early region by alternative splicing, including LT, sT, 57 kT antigen (57 kT) and alternative frame of the large T open reading frame (ALTO). (**C**) LT contains the DnaJ domain with a conserved HPDKGG motif, the MCPyV unique region (MUR) with the retinoblastoma protein (RB) binding motif, the nuclear localization signal (NLS), the DNA or origin binding domain (OBD), the zinc-finger domain (ZN) and the helicase/ATPase domain. sT antigen encompasses the DnaJ domain, the LT stabilizing domain (LSD), and interaction domains for the protein phosphatases PP2A and PP4.

Merkel cell polyomavirus (MCPyV) is a naked double-stranded DNA virus belonging to the *Polyomaviridae* family [1]. Its circular genome of ~5400 base-pairs (bp) encompassed three functional domains (Figure 1). The early region includes the “Tumor” (T) antigen gene locus [2], from which, alternatively-spliced RNA transcripts are produced. This region encodes for distinctive gene products: the large T (LT), small (sT), 57kT antigens and a product from an alternate frame of the LT open reading frame (ALTO) [3]. The LT, sT and 57 kT antigens, due to alternative splicing, share a 78 amino acid sequence at their N-terminal region [4].

Similar to other human polyomaviruses (HPyVs), the MCPyV LT antigen contains a number of motifs and domains that play key roles in viral genome replication and transcription, as well as tumorigenesis (Figure 1). The N-terminal half encompasses the DnaJ domain, which consists of the CR1 motif (13–17 amino acids) followed by the HPDKGG, the sequence is responsible for Hsc70 binding [5,6]. The WXXWW sequence found in LT of other PyVs and that binds the mitotic checkpoint serine-threonine protein kinase Bub1 is absent in MCPyV LT [7]. At this position, MCPyV LT has a sequence known as MCPyV T antigen unique region (MUR), containing a binding motif for the vacuolar sorting protein Vam6p [8]. Adjacent to this, the conserved LXCXE retinoblastoma (RB) binding motif is present.

Finally, a nuclear localization signal (NLS) with sequence RKRK is situated in the N-terminal region of LT [9]. The C-terminal region of LT contains an origin binding domain (OBD) and the helicase/ATPase domain [8]. Both the OBD and the helicase/ATPase domain are required for replication of the viral genome. The C-terminal region of LT of other HPyVs binds to p53, a tumor suppressor that regulates the gene expression in response to events such as DNA damage, leading to apoptosis, cell cycle arrest or senescence, and inhibition of angiogenesis, and is usually deregulated in cancer [10]. This p53 binding site is contained in the OBD and helicase/ATPase domain. The possible p53 binding domain in MCPyV LT and its interaction with p53 is discussed in Section 4.2.

MCPyV-positive MCCs (hereafter referred to as VP-MCC) express a C-terminal truncated LT (tLT) due to nonsense mutations or frameshift mutations generating premature stop codons. Tumor-derived tLTs retain the DnaJ region and the RB binding domain, and sometimes the NLS, but lack the OBD and helicase/ATPase domain [5,11] (Figure 1). The C-terminal region contains several elements fundamental for viral replication, hence tLT fails to support viral replication [12]. As for other HPyVs, and in general for other tumor viruses, there is strong selective pressure within tumors to eliminate viral replication capacity [13].

MCPyV LT is rich in potential phosphoacceptor sites (94 serine, 42 threonine, and 23 tyrosine residues). Li et al., found that phosphorylation of LT at S816 by ATM kinase induced apoptosis and thus contribute to anti-tumorigenic properties of the C-terminal domain [14]. Diaz and colleagues identified three additional phosphorylation sites: T271, T297 and T299. Mutation of T271 into alanine did not have an effect on viral replication. LT T297A stimulated replication, whereas LT T299A was unable to do so. The authors demonstrated that phosphorylation of T297 may negatively regulate viral replication by reducing the binding affinity of LT to the viral origin of replication (ORI), while T299 phosphorylation affects both binding to and unwinding of the DNA [15]. Taken together, truncation of the C-terminal region of LT and phosphorylation of specific residues in LT may abrogate viral replication. S220 is another phosphoacceptor site and the effect of its phosphorylation is discussed in Section 4.1. The phosphorylation status of LT in MCC has not been examined.

As a result of alternative splicing of a common precursor transcript, LT and sT share the 80 N-terminal amino acids [8]. The sT antigen contains the DnaJ domain but lacks the RB motif [16] (Figure 1). At its unique C-terminal region, sT encompasses two zinc-binding domains (CXCXXC motif), which provide structural and functional stabilities and two domains rich in cysteine and proline residues responsible for the sT interaction with protein phosphatase 2A (PP2A) (see further) [17]. A unique MCPyV sT domain, not present in sT of other HPyVs, is the LT stabilization domain (LSD) at residues 91–95. This region, as will be discussed later, is involved in inhibition of proteasomal degradation of LT (Figure 1) [18].

The late region encodes the major capsid protein VP1 and the minor capsid protein VP2 (Figure 1). MCPyV does not seem to express VP3 despite an in-frame internal start codon in the *VP2* gene [19]. When expressed in mammalian cells, VP1 (or VP1 and VP2) self-assemble into 45–55 nm diameter virus-like particles (VLPs) that are used in serological assays [20].

Interspersed between the early and late region is the non-coding control region (NCCR), which contains the ORI characterized by a core of 71-bp sufficient to initiate DNA replication (Figure 1). This core region consists of an AT rich tract and eight 5′-GAGGC-3′ LT binding motifs [12]. The NCRR also contains regulatory elements and bidirectional transcriptional promoters required for early and late viral gene expression [21]. The NCCRs of HPyVs such as BKPyV and JCPyV show often rearrangements that affect viral DNA replication, promoter activity, virus production and could help to increase the pathogenic properties of these viruses [22,23,24]. MCPyV NCCR polymorphism is found, but no specific NCCR architecture seems to be associated with VP-MCC as MCPyV variants with identical NCCR have been isolated from both MCC and non-MCC material [25]. However, MCPyV NCCR variation affects early and late promoter activities in a VN-MCC cell line and in human dermal fibroblast and wild-type LT inhibited both early and late promoter activities in both cell lines, whereas tLT had the opposite effect [25]. A recent study demonstrated the onset of insertions and deletions in the NCCR among an HIV-1-positive population [26]. Whether NCCR variation has an influence on viral replication and pathogenic properties of the virus remains to be investigated.

The molecular characterization of viral genomes has been useful to describe viral lineages associated with specific human populations, as demonstrated for other PyVs [27,28,29]. Phylogenetic analysis, performed on LT and sT antigens and on VP1, showed that MCPyV sequences circulate in Europe/North America, Africa, Asia, South America and Oceania groups, suggesting the occurrence of a viral divergence followed human migrations around the globe [30]. There is a significant evidence for an ancient and relatively stable association of PyVs with their hosts, suggesting that co-divergence is the main factor during the evolution [31]. However, deviations from co-divergence indicate that additional evolutionary processes are at play. Phylogenetic analysis, about the evolutionary history of MCPyV, showed that the MCPyV LT is most similar to gorilla polyomavirus 1 (GgorgPyV1) and chimpanzee polyomaviruses 2 and 3 (PtrovPyV 2 and 3) [1], raising the possibility that MCPyV stems from a nonhuman primate (including chimpanzees and gorillas) and even ape-specific group of PyVs [31]. Non-human primates still represent an important proportion of the bush meat consumed in West and Central Africa (ca. 12%). Hunting and butchering of bush meat provide the major routes of pathogen and a cross-species transmission events (e.g., human immunodeficiency viruses and severe acute respiratory syndrome coronavirus 2). This could also explain how MCPyV may have been transmitted from apes to humans [32].

### 1.2. Seroprevalence

MCPyV prevalence study suggests that this virus is chronically shed from human skin representing part of the skin microbiota [33]. The initial exposure to MCPyV, based on the VP1 serology assay, supposedly occurs in early childhood. As reported in a study from Cameroon, significant titers against MCPyV were detected in newborns, although these titers decreased to undetectable levels by 16 months of age [34]. The maternal derived antibodies could represent the reason of the seropositivity in newborns. Moreover, these antibodies, effective in preventing primary infection, could explain why the seroprevalence is lower in children and higher in adults [34]. By 18 months of age, when the maternal antibodies were no longer detectable, children were susceptible to de novo infection and were able to mount an own antibody response. Beginning at 18 months of age, an increasing fraction of children became positive until approximately 80% tested positive at the age of 5 [34]. In a separate cohort from the same study, the correlation of seropositivity was observed between siblings of similar ages, suggesting that siblings likely were exposed to MCPyV at the same time and by each other [35]. These data suggest that transmission may occur via direct contact with the skin or saliva [34,35]. Several studies support the increasing risk with age for exposure and persistent infection by MCPyV [36,37,38,39]. A study conducted in Italy, with participants aged from 1 to 100 years old, showed how the seroprevalence for MCPyV rapidly increased with age: from 41.7% in children age from 1 to 4 years old, to 87.6% among young adult (15–19 years old), remaining frequent in adulthood (79–96.2%) [40]. MCPyV seroprevalence studies performed in China (61% overall) and the Czech Republic (63%) yielded similar results with an increasing trend with age [41,42]. Antibodies versus MCPyV LT and sT are detected in about 1% of healthy individuals and they can be present in patients with MCC [43]. Often MCC patients have higher titers of VP1 antibodies than normal healthy individuals [20].

### 1.3. Cell Tropism: Skin; Replication in Dermal Fibroblasts

Because MCPyV was originally detected in MCC, a tumor believed to originate from Merkel cells (MCs), which are specialized skin cells, and is chronically shed from skin from healthy individuals, it was believed that the virus is dermatotrophic. It is now questioned that MCs are the target of MCPyV infection or productive replication because there are too few MC in the human skin to account for the millions of copies of MCPyV DNA detected on healthy skin [33]. Liu et al., speculated that the natural MCPyV host cells were one of the more abundant cell types in the human skin. They showed that human dermal fibroblasts support productive viral replication [44], and because MCs are situated in the basal layer of the epidermis near dermal fibroblasts, the authors hypothesized that MCPyV actively replicating in the dermal fibroblasts could accidently enter MCs and cause MCC [44]. Likely, MCs could represent a replication environment that supports viral integration and transformation [44]. It has also been demonstrated that MCPyV is capable of expressing LT and VP1 in fibroblast cell lines originating from lung tissue [44]. Hence, an active viral replication of MCPyV might be connected to all fibroblast tissues [44]. MCPyV DNA has been detected in cutaneous swabs [45] and it is possible that infected dermal fibroblasts might die and virions could be carried to the skin surface by the flow of differentiating keratinocytes [46]. This suggests that viral particles can be more widespread from the site of replication and release. This hypothesis is supported by the observation that MCPyV is frequently detected in eyebrow hair bulbs [47]. MCPyV can infect dermal fibroblasts near hair follicles and it is possible that mature virions could be cleared to the surface of human skin through hair follicles and/or associated sebaceous and sweat glands [47].

## 2. MCPyV and MCC

MCC is a rare, neuroendocrine, cutaneous malignancy that was first described in 1972 by Toker as “trabecular carcinoma of the skin” [48]. The name was later changed to MCC, since the tumor cells were similar to Merkel cells, present in particular around hair follicles and in the basal layer of the epidermis. Although MCC is a rare skin cancer, it is highly aggressive displaying a mortality rate of ~45% [49]. Consequently, MCC has a case-fatality rate higher than observed with melanoma [49]. Almost one third of the patients, at primary diagnosis, present loco regional metastases or lymph node metastases [49]. During the last 10 years, MCC incidence has increased significantly and is expected to increase further, since, the occurrence of this type of cancer, is related with aging (immunosenescence) and exposure to the sun [50]. An important alternative explanation for this finding is that before the large use of CK20 immunostaining, the pathology diagnosis was difficult and may at these ancient times require electronic microscopy, which was frequently not performed. Thus, true MCC were frequently misclassified [51,52]. The correlation between MCC and UV radiation is well documented [53]. Pigmentation of the skin seems to protect against MCC, as black, Asian and Hispanic individuals have considerably lower risk of MCC than white populations. Moreover, the occurrence of MCC is frequent in elderly patients on chronically sun-exposed skin, in individuals treated with UVA photo-chemotherapy and in patients with a history of other skin cancers associated with sun exposure. Melanoma is also linked with a three-fold greater risk of MCC [54]. A molecular UV signature, characterized by DNA mutations that are typically caused by UV damage, such as C to T transitions, has been demonstrated only in a subset of cases of VN-MCCs [55,56]. The association with UV exposure in VP-MCC could be related to other factors, such as UV-induced immunosuppression. In fact, immunodeficiency forms a risk factor in the development of MCC. MCC is more frequent in patients with leukemia [57] or HIV infection [58] and in those who are immunosuppressed, as a result of organ transplantation or other causes [59]. The mortality is higher in immunosuppressed individuals than in immunocompetent patients [60]. These findings emphasize the crucial role of an efficient immune surveillance in the control of tumor growth and progression.

While ultraviolet radiation induced DNA damage is implicated in VN-tumors, the major causative factor of the MCC is considered MCPyV [61]. MCPyV was first identified in 2008, through whole-transcriptome sequencing [62], integrated into the genome of eight out of ten tested MCC cells. The Southern blot patterns of the primary tumor and a metastatic lymph node, isolated from the same patient, demonstrated an identical viral DNA integration at several different chromosomal sites. This important finding indicated that the viral integration was clonal and it was an early, if not initiating event, in VP-MCC oncogenesis process [62]. In addition, a C-terminal tLT form, lacking the OBD and helicase activity of LT required for viral DNA replication, was also observed [62]. Numerous studies have now confirmed that 80% of the examined tumors contain clonally integrated copies of the virus and express tLT [62,63,64]. MCPyV integration into the host genome occurs by accidental genome fragmentation during viral replication, in random site, without involvement of cellular tumor suppressor genes or oncogenes [56]. Viral integration involved mutations that result in the truncation of LT and a study by Schrama and co-workers suggests that truncating mutations occur before or during integration [65]. In vitro cell studies have demonstrated that expression of full-length LT in VP-MCC causes a specific DNA damage response, which is probably induced by in situ replication of the integrated viral DNA, which in turn is triggered by the binding of LT to the MCPyV ORI. Truncation of LT abolishes viral replication and seems to be necessary for MCC oncogenesis [5,66]. Tumor-derived tLT preserves the N-terminal J domain and LXCXE motif, whereas the DNA binding, helicase and cell growth-inhibitory domains are lost [66]. The tLT could potentiate a stable integration of the MCPyV into the host genome [66]. All VP-MCC tested contain ≥1 viral genome copies/cell [65,67,68,69,70], whereas in non-MCC tumors that contain MCPyV, the viral load was at least 2–3 logs lower (reviewed in [61]).

## 3. Cells of Origin of MCC

It was originally proposed that MCC derived from MCs because of similar immunophenotypes [71]. Both cell types express cytokeratin 20 [72], synaptophysin [73], neural cell adhesion molecule/CD56 [74], and numerous endocrine markers [75]. However, it is more and more unlikely that MC are the cells of origin because several characteristics of MCC argue against MC as the progenitor cell of MCC. Epithelial, fibroblastic, lymphoid, and neural crest origin of MCC has been put forward (Arguments in favor or contra these cell types as origin of MCC are summarized in Table 1.

VP-MCC may also originate from different cell types than VN-tumors. Dermal fibroblasts were suggested since they are permissive for MCPyV infection [44], but also keratinocytes could be the cell of origin of VP-MCC because keratinocyte-specific expression of MCPyV oncoproteins resulted in oncogenic effects [76]. Other studies suggest that VN-MCC derive from epidermal keratinocytes, whereas VP-MCC derive from dermal fibroblasts [77,78].

A recent report supports the assumption that VP-MCC may derive from the epithelial lineage [79]. The authors sequenced a combined tumor of trichoblastoma (neoplasm of epithelial follicular germinative cells) and VP-MCC. Non-integrated viral DNA encoding full-length LT could be amplified from the trichoblastoma, while integrated virus (~20 copies/cell) was detected in the MCC. Remarkably, two different tLT may be expressed in this MCC tumor.

Whole genome sequencing identified six somatic mutations common for both tumors. The trichoblastoma had expression of KRT17 and SOX9, and activation of GLI1 as observed by nuclear localization, markers that are shared with MC progenitors [102,103,104]. Therefore, the authors suggest that the trichoblatoma cell in which MCPyV integration occurred and led to the development of MCC could be an epithelial progenitor cell of the hair follicle or an already differentiated MC [79].

## 4. The Oncogenic Mechanisms of MCPyV T Antigens

### 4.1. MCC Cell Growth Depends on LT But Not sT

Since the early proteins of other HPyVs possess oncogenic potentials in cell cultures and in animal models [105], the role of LT and sT in tumor growth was examined. Knock down of sT and LT (i.e., truncated LT and 57kT which cannot be distinguished in most VP-MCC cell lines) reduced MCC cell proliferation in culture, but also in xenograft mice [98,106,107]. Specific knockdown of only LT was sufficient to generate growth inhibition. Rescue experiments, i.e., expression of T antigens in cells where their endogenous expression was knocked down showed that wild-type sT plus LT could rescue cell growth. The growth promoting property of LT involves binding to RB because mutations in the DnaJ domain, the RB domain, or S220A abrogates LT’s ability to promote cell growth [106,108,109].

Ectopic expression of the tLT variant MKL-1 in MCC13 promoted cell cycle progression [109]. However, RNA interference studies showed that sT is dispensable for growth and survival of VP-MCC cell lines [98]. Interestingly, knockdown of the T antigens in the VP-MCC LoKe cell line did not results in any growth inhibition. The authors speculate that additional aberrations enable cell growth even in the absence of T antigens and therefore, in some VN-MCC cases a viral hit-and-run mechanism was possible where MCPyV initiates tumor formation, then disappears, but additional mutations drive tumor progression and maintenance [110]. Studies in mouse and human fibroblasts demonstrated that expression of a tumor-derived tLT has stronger growth promoting activities than wild-type LT and 57kT [111]. Expression of full-length did not induce anchorage-independent growth, whereas tLT proteins induced aggregates in soft agar that did not grow into full colonies, suggesting that tLT has increased cell proliferative capacity compared with the wild-type LT. Expression of the C-terminal 100 amino acids residues inhibited the cell growth of fibroblasts and of the VP-MCC MKL-1 cell line [111]. The mechanism by which this region inhibits cellular growth is not known but is likely to be independent of p53 since neither full-length LT nor 57 kT are able to bind p53. The C-terminal domain may interact with a yet unidentified cellular protein involved in growth regulation. Putative candidates are the cell cycle checkpoint kinase ATM, casein kinase 2β and phosphatidylinositol-5-phosphate 4-kinase type 2β, which are all involved in proliferation and were found to interact with MCPyV LT, but the biological importance of these interactions were not examined, nor was the region of LT required for interaction identified (reviewed in [21]). CRISPR/Cas9 targeting of LT/57kT impaired MS-1 and WaGa cell proliferation, decreased G1/S cell cycle progression and increased apoptosis. Additional targeting of sT did not enhance the effect in LT/57kT mutated cells [112].

### 4.2. Oncogenic Properties of LT

Cell culture studies revealed that neither full-length nor tumor-derived tLT was able to trigger cellular transformation [99], but LT is required for growth of VP-MCC cells [108,113]. The C-terminal domain of LT causes DNA damage and stimulates host DNA damage response, leading to p53 activation and inhibition of cellular proliferation. Phosphorylation of the C-terminus by ATM kinase induces apoptosis and inhibits proliferation [14,66]. Thus, the C-terminus of LT contains anti-tumorigenic properties and may explain why this region is deleted in VP-MCC. To further elucidate the role of MCPyV LT in MCC tumorigenesis, cellular proteins that interact with LT were identified using different methods [21,114]. However, the biological relevance of these interactions and possible implications for MCPyV-induced cancer have not always been studied.

#### 4.2.1. LT and p53

LT expressed in VP-MCC is truncated in its C-terminal part, which encompasses the p53-binding domain in LT of other HPyVs. As expected, tLT did not interact with p53, but surprisingly neither did full-length LT [111]. In another study, Borchert and co-workers showed that an antibody against p53 could immuno-precipitate full-length, but not tLT [115]. However, LT did not bind p53 directly and LT, but not tLT inhibited p53-mediated transcription. They suggested that full-length LT interacts with a bridging protein that serves as a co-activator in p53-driven transcription. Alternatively, another protein may change the conformation of LT allowing it to bind p53 as has been shown for human papillomavirus E6 protein. E6 forms a complex with E6AP and p53, but neither E6 nor E6AP are separately able to recruit p53. However, E6AP renders the conformation of E6 competent for interaction with p53 [116]. Park et al., reported that expression of tLT in IMR90 lung fibroblasts significantly stimulated transcript levels of p53-responsive genes and increased both total protein and Ser-15 phosphorylation levels of p53 [117]. They showed that the interaction between LT and RB1 lead to increased levels of ARF and activation of p53. ARF is an inhibitor of the E3 ubiquitin protein ligase MDM2, which degrades p53 [118]. Hence, LT can through RB-ARF-MDM2 axis stabilize p53.

#### 4.2.2. LT and Retinoblastoma (RB) Family

Both full length and tLT interact with RB1, although with different strength [111,115,117]. This suggests that LT may usurp RB1, thereby relieving repression of E2F-mediated transcription and induce cell cycle progression into S phase. MCPyV LT did not interact with the p107 and p130 retinoblastoma family members, nor did it interfere with p107-induced and p130-induced cell cycle arrest and repression of E2F responsive genes [111,113,115]. The weaker in vitro oncogenic potentials of MCPyV LT compared to LT of other PyVs may be attributed to its weaker impact on the tumor suppressors p53 and RB.

#### 4.2.3. LT and HSC70

LT interacts with HSC70 via the DnaJ domain and stimulates viral replication [12]. Like other PyVs, it is presumed that MCPyV LT disrupts Rb-E2F family complexes through the action of its DnaJ domain and ATPase activity of Hsc70 [119,120]. The biological significance of the DnaJ domain in sT is unknown as mutations in DnaJ of sT did not interfere with its effect on viral replication or in vitro transformation activity [12,99].

#### 4.2.4. LT and VPS39 Subunit of HOPS Complex/Vam6p

Human Vam6p, a cytoplasmic protein involved in lysosomal processing and clustering, interacts with MCPyV full-length LT as well as MCC-derived tLT [18]. LT and tLT that retains its nuclear localization signal translocate hVam6p to the nucleus and sequester it from involvement in lysosomal trafficking. The physiological consequences of LT:Vam6p interaction are not known, but it might play a role in MCPyV replication rather than tumorigenesis, because VP-MCC have been described that express tLT without a nuclear localization signal [62,121,122].

#### 4.2.5. LT and ATOH1

Sox2 (sex-determining region Y-box 2) and Atoh1 (atonal homolog 1) are critical transcription factors for MC development in mice [123]. Harold and colleagues found that knockdown of all T antigen isoforms in VP-MCC cell lines co-cultured with human keratinocytes promotes a neuronal phenotype in the MCC cells and resulted in reduced expression of ATOH1 and SOX2 [124]. The tLT 339 variant stimulated ATOH1 and SOX2 expression levels, but neither a LT399 retinoblastoma binding deficient mutant nor sT increased expression of ATOH1 and SOX2. Activation of the SOX2-ATOH2 pathway by LT in a retinoblastoma-dependent manner is important for both the manifestation of a Merkel cell phenotype and tumorigenesis. Transcriptional activation by ATOH1 requires E-boxes (5-CANNTG-3′) and E47 binding site [125], both of which are present in the miR-375 promoter. Indeed, ATOH1 stimulated expression of miR-375 and ectopic expression of tLTs stimulated the activity of a minimal promoter containing three E-box and induced *ATOH1* mRNA and miR-375 in fibroblast MRC-5 cells [126]. Moreover, high transcript levels of *LT* and *ATOH* were detected in the VP-MCC WaGa cells. sT, however, was unable to enhance *ATOH1* mRNA and miRNA-375 levels. The neuroendocrine features of MCC may therefore be linked to MCPyV-induced expression of ATOH1. Whether LT-induced expression of miR-375 is exclusively mediated by ATOH1 or also by an ATOH1-independent mechanism remains to be elucidated. As both *ATOH1* and miR-375 promoters were hypomethylated, LT may stimulate demethylation of these promoters. Finally, strong expression of ATOH1 and miR-375 was also observed in classical VN-MCC cells, indicating a virus-independent mechanism in their expression [126].

#### 4.2.6. LT and Ubiquitin-Specific Protease 7 (Usp7)

All MCPyV T-antigens interact with Usp7, a cellular deubiquitination enzyme [127]. The binding with LT, tLT and 57kT is direct, whereas sT probably interacts indirectly. Binding of Usp7 required the tumor necrosis factor receptor-associated domain of Usp7 and did not alter the ubiquitination levels of the T antigens, but stimulated the binding affinity of LT to the ORI, thereby restricting viral DNA replication. Usp7-mediated restriction of MCPyV replication could promote viral persistence [127]. Whether Usp7:T antigens interaction contributes to MCC tumorigenesis remains elusive. However, interference with other functions of Usp7 such as DNA damage response, epigenetic regulation, and immune response may also play a role in the development of virus-induced MCC [128].

#### 4.2.7. LT and Other Interacting Proteins

Other interaction partners of MCPyV LT are summarized in Table 2. The interaction in VP-MCC has not been validated and the biological consequences of these interactions have not been investigated.

Autophagy plays an important role in cancer and in immune evasion [141,142,143]. Silencing LT or LT+sT in VP-MCC cell lines reduced the expression of miR-30a-3p, miR-30a-5p and miRNA-375, while ectopic expression of tLT or sT in VN-MCC cells increased the levels of these miRNAs. Induced expression of miR-30a-3p, miR-30a-5p and miRNA-375 required the DnaJ domain [144]. Target mRNA of these miRNAs encode the autophagy proteins ATG7, SQSTM1/p62 and BECN1. The authors showed that sT and tLT, but not wild-type LT suppressed autophagy processes in MCC cells and protein levels of ATG7 and SQSTM1/p62 were lower in VP-MCC compared with VN-MCC. Hence, T antigens-mediated suppression of autophagy might protect cancer cells from cell death and contribute to tumorigenesis [144].

### 4.3. The Role of 57kT and ALTO in VP-MCC

Whether 57 kT and ALTO are implication in MCPyV-induced tumorigenesis remains to be established. The 57kT protein retains the RB binding domain and the CR1 and DnaJ binding motifs. Immortalized human fibroblasts Bj-hTERT expressing 57kT grew slower than control cells and when LT cDNA was stably expressed in mouse and human fibroblasts, the 57kT form was preferentially expressed. Expression of 57 kT has never been detected in VP-MCC [68,69], but due to truncation in the LT gene, LT and 57kT cannot be distinguished in most MCC using the antibodies currently available. The role of 57kT in MCC remains unsolved. Deletion of ALTO did not abrogate viral replication and is dispensable for MCPyV-driven tumor cell proliferation [3,108], but the function of this protein remains elusive.

### 4.4. The Oncogenic Properties of sT

MCPyV sT is sufficient to fully transform Rat-1 and NIH3T3 mouse fibroblasts [98,99,100]. Knockdown of sT expression in VP-MCC cell lines causes cells to stop proliferating, but did not result in cell death. Co-expression of full-length or tLT did not enhance sT-induced colony formation compared with expression of sT alone [99].

#### 4.4.1. sT and Transgenic Mice

Considering the non-transforming potentials of LT in cell culture and that sT can induce transformation, sT, but not LT transgenic mice models have been generated. Verhaegen et al., generated a transgenic mouse model in which sT expression was regulated by the epidermis-specific keratin-5 promoter [134]. Analysis of embryos revealed that sT promotes neoplastic transformation in epithelia in a PP2A-independent, but LSD-dependent manner. Adult animals developed lesions strongly resembling squamous cell carcinoma in situ. However, expression of sT alone does not appear to be sufficient to drive epidermal cells in MCC in a mouse model. The same group generated K5-tLT, K5-sT+tLT, K5-st+Atoh1, K5-tLT+Atoh1, K5-sT+tLT+Atoh1, and K5-Atoh1 transgenic mice [145]. The tLT embryo had no apparent phenotype, co-expression of sT+Atoh1 resulted in MCC-like tumors, and co-expression of tLT did not noticeably altered the phenotype of sT or sT+Atoh1 mice. These studies indicate that Atoh1-induced differentiation of epidermal cells into neuroendocrine lineage together with sT as the viral oncogenic driver can result in MCC development. Transgenic mice co-expressing sT and tLT under control of the keratinocyte-specific K14 promoter developed hyperplasia, hyperkeratosis and acanthosis, and some mice develop papillomas, but not MCC [76]. Shuda and c-workers developed a sT-Δp53-Atoh1 transgenic mice which allowed sT expression in MC cells [89]. Although these mice have increased embryonic MC precursor proliferation, they did not develop MCC.

Taken together, in vitro and animal studies and the detection of sT in the absence of LT in some VP-MCC indicate that sT may be more involved in the oncogenic process, whereas LT is required to maintain the tumor cell growth [98,99]. However, studies in the genuine cells of origin of VP-MCC are required to determine the requirements of sT and LT in cell growth and oncogenesis.

#### 4.4.2. sT and Eukaryotic Translation Initiation Factor 4E Binding Protein (4E-BP1)

Transcription initiation factor 4E-BP1, a downstream target of the Akt-mTOR pathway, binds in its unphosphorylated or hypo-phosphorylated form eukaryotic initiation factor 4E (eIF4E), thereby preventing assembly of eIF4F onto capped mRNA and inhibiting translation [146]. sT interacts with 4E-BP1 and expression of sT, but not LT promoted 4E-BP1 phosphorylation [99,133]. sT-induced phosphorylation of 4E-BP1 is accomplished by sT interacting with Cdc20 and possibly Cdc20 homolog 1 (Cdh1), which activates the CDK1/cyclin B1 complex and CDK1 and phosphorylate 4E-BP1 [132,133]. 4E-BP1 hyperphosphorylation was required for sT-induced transformation of rodent cells [99,133]. The importance of sT-mediated 4E-BP1 phosphorylation in MCPyV-induced MCC is not completely understood, but sT-induced hyperphosphorylation of 4E-BP1 can dysregulate cap-dependent translation, an event that has been shown to promote tumorigenesis [147].

#### 4.4.3. sT and E3 Ubiquitin Ligases

Binding of sT to E3 ubiquitin ligase complex SCF^Fbw7^ led to inactivation of the enzymatic activity and stabilization of LT, which is a substrate of SCF^Fbw7^ [135]. Binding occurs through LSD and loss of net positive charge in the LSD abrogated sT:SCF^Fbw7^ interaction [100]. sT-induced stabilization of LT stimulates viral replication and transformation of rodent fibroblasts cell cultures by sT is SCF^Fbw7^-dependent [100,148], and increased protein levels of SCF^Fbw7^ substrates Mcl-1, c-Jun, mTOR and cyclin E in sT transgenic mice [134]. sT also interacts with the E3 ubiquitin ligases Cdc20-anaphase promoting complex [17] and β-TrCP [149] and this stimulated genome instability [135]. Inactivation of E3 ubiquitin ligases by sT may be therefore be an important contributor in MCPyV-induced transformation and tumorigenesis. However, Dye and colleagues failed to detect interaction between sT and SCF^Fbw7^ and sT and β-TrCP and no increased c-Myc levels were observed when sT was overexpressed. They also demonstrated that sT-mediated stabilization of LT did not require SCF^Fbw7^ [150]. The reason for the discrepancies between the different students is presently unknown. sT can form a complex with the E3 ubiquitin ligase STIP1 homology and U-box containing protein 1 (STUB1) [129]. This E3 ubiquitin ligase plays also a role in innate and adaptive immunity [151], but the biological implications of sT:STUB1 interaction in MCPyV replication and MCC remain to be determined.

#### 4.4.4. sT and N-myc Downstream Regulated Gene-1 (NDRG1)

Stable expressing the entire MCPyV early region in human immortalized keratinocytes resulted in >1.5-fold up- or down-regulated of 325 genes [152]. Of these, 73 had decreased expression and the majority encodes proteins involved in cell senescence, DNA repair, signal transduction, and cell cycle regulation, including HIST1H1C. Upregulation of HIST1H1C was also confirmed in VP- and VN-MCC cell lines, MCC tumors, and in sT expressing human fibroblasts expressing compared with normal fibroblasts [153,154,155]. Of the upregulated genes, many encode proteins implicated in cell cycle regulation and signaling pathways, including CDK4, cyclins D2 and D3, CDC25, FOXQ1, DUSP10, and CTSH. One gene that was specifically down-regulated by MCPyV, but and not by other HPyVs and SV40 was the *N-myc downstream regulated gene-1* (*NDRG1*). NDRG1 is a known tumor suppressor and metastasis suppressor [156]. Knock-down of sT+LT in MKL-1, MKL-2, MS-1 and CVG-1 increased NDRG1 levels in all four cell lines, and decreased cyclin D1 and CDK2 levels in MKL-2, MS-1, and CVG-1 cells. Overexpression of NDRG1 in MKL-2 reduced cyclin D1 and CDK2 levels, but not in MKL-1 cells. The different status of transformation of may explain the difference between MKL-1 and the other VP-MCC cell lines. Depletion of sT alone or sT+LT resulted in comparable increase in *NDRG1* mRNA levels, suggesting that sT is sufficient. Overexpression of NDRG1 in keratinocytes stably expressing MCPyV early region or in MKL-1 and MKL-2 cells inhibits cellular proliferation and migration. Taken, together these observations indicate that MCPyV-mediated repression of NDRG1 participates in MCC tumorigenesis and that sT may be the main contributor. The expression levels of NDRG1 have not yet been examined in VN- and VP-MCC. In a study in 91 MCC tumors (30 VN and 61 VP), cyclin D1 expression was only detected in two tumors, both of which were MCPyV negative [157].

#### 4.4.5. sT and p53

LT indirectly activates p53 (see above) and sT can stabilize LT, yet co-expression of LT and sT reduced p53 activation [148]. MCPyV sT can inhibit p53 activity indirectly by binding to and activating the transcription factor MYCL and the histone acetylase complex EP400 [117]. The MYCL:EP400 complex controls transcription of *MDM2* and *CSNK1A1* genes. The latter encodes casein kinase 1α which activates MDM4, an inhibitor of p53 [158]. The activation of p53 by LT may exert anti-tumorigenic effect, while sT-mediated inhibition of p53 favors pro-tumorigenesis. The relative concentrations of LT and sT, but also the strength of impact of LT and sT on p53 will determine the outcome. VP-MCC cells have been shown to express high levels of MDM4 [117]. Accordingly, p53 levels were found to be lower in VP-MCC cell lines compared to VN-MCC cell lines [117,159,160]. Examination of MCC revealed that mutations in *TP53* gene are almost exclusively detected in VN-MCC, but only 7% of VP-MCC expressed detectable p53 levels and an inverse correlation between p53 expression and viral DNA copy number was observed [157,161]. One study reported that p53 levels were variable between patients, with no obvious differences between VN- and VP-tumors [162]. The expression levels of MDM4 in VN- and VP-MCC biopsies have not yet been examined. Another consequence of the interaction of sT with MYCL:EP400 complex that may be involved in tumorigenesis was recently published. This complex stimulates the expression of components of the lysine-specific histone demethylase 1 (LSD1) complex that acts as a transcriptional repressor [117]. Treatment of VP-MCC cell lines with LSD1 inhibitors completely blocked colony formation in soft agar, and LSD1 inhibitors reduced the growth of MCC in vitro and in xenograft models using VP-MCC cells. Hence, sT-mediated activation of the LSD1 complex seems to play a pivotal role in VP-MCC, and LSD1 inhibitor could be used to treat VP-MCC patients.

#### 4.4.6. sT and Protein Phosphatases

Because aberrant or loss of enzymatic activity of protein phosphatases (PPs) can lead to transformation and their role in cancers, PPs are considered tumor suppressors and are targeted by several tumor viruses [163,164,165,166,167]. MCPyV sT interacts with PP1A, 1B and 1G [17,129]. The biological consequences of sT:PP1 interaction have not been determined, but RB is a PP1 substrate. Inhibition of PP1 by sT may therefore results in hyperphosphorylation of RB, release of repression of E2F target genes, and drive to enter the S-phase [168].

PP2A exists as a heterotrimer composed of a structural subunit A, a regulatory subunit B, and a catalytic C subunit [169]. MCPyV sT binds the structural subunit Aβ and Aα, and the catalytic subunits Cα and Cβ. This binding reduced the catalytic activity of the enzyme [17,136,137,138]. sT’s binding to PP2A excluded the regulatory subunit B56α, but not other B subunits [17]. The biological implications of the sT:PP2A interaction are not known because mutations in sT that prevented PP2A binding had no effect on sT’s transforming activity [99], nor did it impede sT-induced skin hyperplasia in transgenic mice [134].

MCPyV sT was reported to interact with PP4 [17,136,139,140], and this interaction promotes microtubule destabilization and stimulates cell motility and filopodium formation [139,140]. The sT:PP4 association also interferes with the NFκB pathway. The transcription factor NFκB is retained in an inactive state in the cytoplasm through interaction with inhibitor of κB (IκB). Activation of the NFκB pathway occurs after phosphorylation of IκB by IκB kinase (IKK) and subsequent degradation of IκB. IKK is a trimeric complex that consists of IKKα, IKKβ, and IKKγ or NFκB essential modulator (NEMO). Release of NFκB allows nuclear translocation where it affects transcription of NFκB-responsive genes [170]. NFκB target genes encode proteins involved in inflammation, immune responses, including antiviral response [171,172]. Griffith and colleagues demonstrated that sT associates with a PP4R1-PP4C complex, which stimulates the interaction between NEMO and the protein phosphatase PP4C-PP4R1 complex. Consequently, NEMO-mediated recruitment of PP4C to the IKK complex reduces IKK phosphorylation, with subsequent inhibition of IκB and failure to release, activate (phosphorylate), and translocate NFκB to the nucleus [136]. Thus, MCPyV may affect inflammatory and immune responses by interfering with the NFκB pathway. However, the importance of the sT:NFκB interaction in tumorigenesis is questioned because a significantly higher expression of pSer-536 RelA/p65 subunit of NFκB was observed in VP- (*n* = 24) compared to VN-MCC (*n* = 17). The phosphorylated p65 form was exclusively detected in the nucleus [173].

#### 4.4.7. sT and Sheddases

MCPyV sT stimulates expression of the sheddases ADAM10 and 17, proteins involved in cell signaling, inflammation, and tumor formation and progression [174]. The exact mechanism by which sT enhances ADAM 10 and ADAM17 expression is not known, but sT increases expression of the transcription factors ACAD8, PPARG, and ITGB3BP that activate the ADAM10 promoter [155]. ADAM 10 and 17 protein levels are higher VP-MCC tumors compared to VN-MCC, suggesting that sT-induced sheddase expression may contribute to MCC progression [174].

#### 4.4.8. sT and Metabolism

Ectopic expression of sT in normal human fibroblasts IMR90 resulted in significantly perturbed metabolism with elevated aerobic glycosylation and upregulation of transcription of metabolite transport genes [155]. Proteins whose transcripts were significantly upregulated included monocarboxylate lactate transporter 1 (MCT1), glucose transporter GLUT1, and GLUT3. Inhibition of MCT1 activity suppressed the growth of VP-MCC cell lines and impaired MCPyV-dependent transformation of IMR90 cells. The authors showed that MYCL cooperates with the tumor derived MCPyV early region (expressing sT and tLT) to induce expression of MCT1 and knockdown of the p65 subunit of NFκB reduced sT, as well as sT+MYCL stimulated MCT1 expression. Taken together, these data suggest that sT-mediated changes in the metabolic state are implicated in virus-induced MCC tumorigenesis. MCT1 expression levels in VN- and VP-MCCs have not been examined, but inhibitors of MCT1 could be considered to treat VP-MCC.

#### 4.4.9. sT and Other Interaction Partners

Other cellular proteins reported to interact with MCPyV sT are shown in Table 2. The interaction in genuine host cells for MCPyV and in VP-MCC has not been confirmed, nor has the physiological relevance of these interactions been explored.

### 4.5. Effect of MCPyV on Signaling Pathways in MCC

#### 4.5.1. The Phosphatidyl-3-Kinase/AKT/Mammalian Target of the Rapamycin (PI3K/AKT/mTOR) Pathway

The PI3K/AKT/mTOR pathway, which plays pivotal roles in cell growth, motility, survival, metabolism, and angiogenesis is often the target of viral infections [175,176]. Strong staining with phosphoT308 AKT antibodies was observed in most of the MCC samples examined, but there was no significant correlation between phosphoAKT and MCPyV status [177,178]. Another study reported AKT phosphorylation in 4 VN-MCC cell lines, but not in VP-MCC cell lines [179]. However, three of the tested VN-MCC cell lines (MCC13, MCC26 and UIOS) are non-classical MCC cell lines High expression levels of PI3Kα and PI3Kδ were observed in respectively 20% and 52% of archival MCC specimens (*n* = 50) [180]. The viral status in the MCC samples was not described, but PI3Kα transcript levels were detected in 2 VN and 2 VP-MCC cell lines, while one of the VP-MCC cell lines (MKL-1) had no detectable PI3Kδ mRNA levels. This suggests that the expression levels of PI3K do not depend on the presence of MCPyV, which is underscored by the finding that silencing of LT and sT in four MCPyV positive MCC cell lines had no effect on AKT phosphorylation [177]. Taken together, the results indicate that activation of AKT in MCC is not caused by MCPyV. A well-known substrate of the PI3K/AKT/mTORC1 pathway is 4E-BP1 and its interaction with sT was discussed earlier.

#### 4.5.2. Protein Kinase C Pathway

Protein kinase C (PKC) is family of serine/threonine kinases that comprises PKCα, βI, βII, γ, δ, ε, η, θ, ζ and ι [181]. Because PKCε plays critical roles in cancer [182], its activation (i.e., phosphorylation of Ser729) was examined in 8 VP-MCC and three VN-MCC specimens [183]. Seven of the VP-MCCs were positive for phospho-PKCε, whereas only one of the three VN-MCC samples expressed phospho-PKCε. These results suggest a correlation between PKCε activation and MCPyV positivity in MCC. However, relative few samples were examined and the involvement of MCPyV in PKCε activation remains to be proven.

#### 4.5.3. Notch Pathway

There are four human Notch receptors (NOTCH1-4) and their ligands include Jagged 1 and 2, and Delta-like proteins [184]. Relative expression levels of NOTCH1, NOTCH2, NOTCH3, and Jagged 1 were compared in 19 VN- and 19 VP-MCC tumors [185]. NOTCH3 expression was higher in VP-MCC compared to VN-MCC, while the opposite was found for Jagged 1. Patients with higher NOTCH3 expression had better overall survival, whereas expression of NOTCH1 and NOTCH2 was not associated with MCPyV status or prognosis. Whether MCPyV proteins are implicated in the upregulation of NOTCH3 and downregulation of Jagged 1 remains to be investigated. MCPyV sT can bind NOTCH2, but the functional implication of this interaction is not known [129]. sT may also activate the NOTCH pathway through stimulating the expression of ADAM10 [174].

#### 4.5.4. Hedgehog Signaling Pathway

Patched 1 (PTCH1) is the receptor for the hedgehog ligand of which 3 are found in humans: sonic (SHH), Indian (IHH), and desert (DHH) hedgehog [186]. Expression of SHH and IHH was monitored in 29 VP-MCCs and 21 VN MCCs. A significant higher expression of SHH and IHH was observed in the VP-MCCs than in VN-MCCs [187].

#### 4.5.5. Apoptotic Pathway

Expression of pro-survival proteins Bcl-2, BclX_L_, Bcl-w, Mcl-1 and A1 has been investigated in both VN- and VP-MCC. High expression of these anti-apoptotic proteins was measured in most MCC and no correlation was found with the viral status of the tumor [159,188,189,190,191,192]. Despite high Bcl-2 levels in most tumors, a phase II clinical trial with Bcl-2 antisense RNA G3139 showed very little efficacy in 12 MCC patients [193].

## 5. Immune Evasion of VP-MCC

More than 90% of the MCC patients are immunocompetent and VP-MCC tumors are highly antigenic, yet they evade immunological destruction [57,63,194]. MCPyV can escape detection by the immune system by different mechanisms. Down-regulating major histocompatibility complex class 1 (HLA class 1) was observed in 84% of MCC tumors, and HLA class 1 expression was significantly lower in VP-MCC than in VN-MCC [195]. MCPyV-specific T cells and MCC-infiltration lymphocytes express elevated levels of multiple markers of exhaustion such as programmed death 1 (PD-1) and T cell immunoglobulin and mucin domain-containing protein 3 (TIM-3) [196]. Moreover, the level of vascular E-selectin is reduced in >50% of the examined MCC (*n* = 56; viral status not determined) and this negatively affects the ability of lymphocytes to migrate into the tumor microenvironment [197]. Programmed death ligand -1 (PD-L1) may be aberrantly expressed by tumor cells, creating a shield against immune attack [198]. Immunohistochemical staining of 8 VN-MCC and 34 VP-MCC showed that none of the VN tumors expressed PD-L1, while 50% of the VP-MCCs were positive for PD-L1 [199]. Another study on 14 MCC (6 VN and 8 VP) reported that 1 VN-MCC had few (1%) PD-L1 positive tumors cells, whereas 7 of the VP-MCC were PD-L1 positive with 2–7.5% of the cells expressing PD-L1 [200]. It is not known whether MCPyV can affect the expression of PD-L1, but upregulation of PD-L1 has been observed in persistent infection with the oncoviruses hepatitis B and C [201].

MCPyV can also avoid the innate immune system because its early region downregulated the expression of TRL9 in the B lymphocyte RPMI-8226 cell line by targeting the transcription factor C/EBPβ [202]. sT could also reduce TLR9 expression, but the mechanism is not known, but it may operate by stabilizing LT [148]. A study on 128 MCC patients revealed that decreased expression of TLR 9 correlated strongly with MCPyV positivity of the tumor, while expression of TLR2, 4, 5, and 7 did not correlate with the viral status of the tumor [203].

The interference with the NFκB pathway by MCPyV sT was discussed earlier. However, another putative mechanism by which MCPyV can interfere with this pathway is through the interaction of LT with bromodomain protein 4 (Brd4). Brd4 acts as a transcriptional and epigenetic regulator [204], and can interfere with the NFκB pathway by interacting with IκB [205]. Brd4 stimulates MCPyV DNA replication by interacting with MCPyV LT and recruitment of replication factor C [130]. Arora et al., showed that also tLT binds Brd4 and that co-expression of Brdr4 in combination with either LT, sT, or tLT did not stimulated MCPyV promoter activity in U2-OS cells [131]. However, Brd4 in combination with LT+sT, but not with tLT+sT, enhanced promoter activity. Studies by our group showed that full-length LT inhibiting the activity of early as well late promoter from 8 different MCPyV variants in MCC13 and immortalized human dermal fibroblasts, whereas truncated variants stimulated their cognate promoter in both cell lines. The effect of sT on MCPyV promoter activity was not examined [25]. The study by Arora and colleagues was done in U2-OS cells and it was not specified if early or late promoter activity was monitored and from which virus strain the promoter was derived. Moreover, they used tLT referred to as tLT21 and tLT168, while we used tumor-derived tLT and tested their effect on the corresponding promoter. Whether the MCPyV LT:Brd4 interaction interferes with NFκB signaling pathway and contributes the virus-induced tumorigenesis remains to be investigated.

Cytokines trigger inflammatory and immune responses upon viral infection [206,207], and play a pivotal role in tumorigenesis [208,209]. A study in BJ human foreskin fibroblasts showed that tLT or tLT+sT induced IL-1β, IL-6, IL-8, and CXCL1 levels, but their expression levels have not been monitored in VN- and VP-MCC cell lines or tumor tissue [210]. Prokineticins are chemokine-like proteins that possess angiogenic and immunoregulatory activities [211]. VP-MCCs had higher prokineticin-2 mRNA levels than the virus-negative tumors [212]. Our group found that chemokine (C-C motif) ligand 17/thymus and activation-regulated (CCL17/TARC) is upregulated in VP-MCC cell lines compared to VN-MCC cell lines. Full-length and tLT, but not sT, enhanced the CCL17/TARC promoter activity and increased protein levels [213]. The exact mechanisms by which MCPyV may affect cytokine expression and their possible role in MCC remain to be determined. Another study reported that sT downregulates IL2, IL-8, CCL20 and CXCL9 expression in the VN-MCC cell MCC-13 [136], but expression levels in VN- and VP-MCC tumors have not been compared. Stimulator of interferon genes (STING) is a signaling molecule that controls type I interferon and other proinflammatory cytokines production [214]. STING protein was undetectable in VP MKL-1, MKL-2 and MS-1 cells, but not in non-classical VN MCC13, MCC26 and UISO cells. Five MCC tumors (virus state not mentioned) also stained negative with STING antibodies [215]. STING silencing may help MCC tumor cells to escape immune eradication. More VN- and VP-MCC should be scrutinized to establish whether STING is specifically silenced in the VP-MCC and the potential role of T antigens in silencing STING should be explored. Postsurgical adjuvant radiation is common in the treatment of MCC patients and although adjuvant radiotherapy can improve locoregional control with reduced recurrence rate of the tumor, it may not affect overall survival [216,217,218,219]. Whether VN- and VP-MCC patients display different sensitivity to radiation is not known, but a previous study had shown that absence of STING impaired radiation-mediated tumor regression [220]. Because it was recently reported that STING expression is downregulated in VP-MCC cells [215], VP-MCC may be less sensitive to radiotherapy than VN-MCC.

## 6. Specific Biomarker for VP-MCC

Apart from detection of viral DNA, RNA, and protein, diagnostic markers that specifically discriminate VP-MCC from VN-MCC are lacking. Likewise, biomarkers to predict disease progression and response to therapy of VP-MCC are lacking. However, the presence of antibodies against LT and sT may be used a diagnostic and prognostic marker. While most individuals have antibodies against MCPyV VP1 (see Section 1.2), only ~1% of healthy patients had low titer antibodies against viral T antigens, whereas 41% of MCC patients had such antibodies [221]. The viral status of all MCC patients was not known, but for those patients it was known, serology for the LT/sT much more closely reflected the virus status of the tumor. In addition, the titers of T antigens antibodies decreased rapidly in patients whose cancer did not recur, whereas they rose with disease progression. So antibodies against LT/sT can predict if the patient has a VP tumor, but these antibodies can also be used to monitor the development of the disease and whether the patient respond to treatment or not.

Some putative markers will be discussed in this section, although most of them do not seem to be very specific and more VN- and VP-MCC patients need to be studied to validate their usefulness.

Several studies have shown that p63 may be an adverse prognostic factor as high levels have been linked to a worse prognosis [222,223,224,225], but the viral status in the MCC tumors was not always described. In one follow up study, the presence of MCPyV was examined, but no correlation between p63 expression and viral presence was found [226]. The chromatin architectural factor DEK was found to be expressed in 15/15 MCC tumors examined, but the viral presence or the clinical stage of the tumors was not identified [227]. This protein is also overexpressed in other cancers (reviewed in [228]), so that it is not a specific MCC biomarker. K homology domain-containing protein overexpressed in cancer (KOC=IMP3) is overexpressed in 90% of the MCC samples (*n* = 20) and expression correlated with metastasis, but the relationship with MCPyV was not investigated [229]. KOC is a prognostic marker in pancreatic cancers and melanomas [230,231] and might be a prognostic marker for MCC. Other proteins examined in MCC include vitamin D receptor, the inhibitory ligand of the Notch receptor Delta-like protein 3 (DDL3), HIF-1α and its target genes GLUT-1, MCT4, CAIX, and vascular endothelial growth factor receptor 3 (VEGFR-3), and P-cadherin [232,233,234,235,236,237,238]. However, the viral status of the tumor was not known (vitamin D receptor), no difference between VN- and VP-MCC was found (GLUT-1, MCT4, CAIX), or there was a tendency to higher expression in VP-MCC, but the difference was not significant (DDL3, HIF1α, P-cadherin). VEGFR-3 was found in all MCCs, but significantly higher in VP-MCC [238]. The value of VEGFR3 as a biomarker is controversial because other studies failed to detect VEGFR-3 in MCC [232,233]. The inconsistency, lack of virus status and limited number of samples of these studies have failed to identify a bona fide biomarker for VP-MCC.

### MicroRNA as VP-MCC Biomarkers

MicroRNA (miR) are small RNA molecules that inhibit gene expression at a post-transcriptional level by preventing translation or inducing degrading of their target mRNA. Because of their stability, presence in all body fluids, and sometimes disease-specific expression, they can be useful prognostic and diagnostic markers in cancer. Several groups have examined miR expression in MCC (reviewed in [239]), but miR-375 in particular has been more extensively studied. This miR is enriched in VP-MCC compared to VN-MCC [240], while another study found that the miR-375 levels were enhanced in MCC cell tumors but not associated with the viral status [241,242]. The MCPyV genome itself encodes a single miR (mcv-miR-M1) that is complementary to a sequence in the *LT* gene adjacent to the RB binding motif. However, mcv-miR-M1 was detected in ~50% of 38 tested VP-MCC and the expression levels were low [243]. It seems unlikely that mcv-miR-M1 contributes to MCVPyV-induced tumorigenesis and its use as biomarker for VP-MCC is doubtful.

## 7. VP-MCC Specific Therapy

Current MCC treatment include surgery, radiation therapy, chemotherapy and immunotherapy. The standard option is surgery followed by radiation [63,64,244]. However, immunotherapy-based strategies is rapidly becoming a preferred therapy in several cancers, including MCC. Three types of immune checkpoint inhibitors are currently being applied on MCC patients: pembrolizumab and nivolumab, antibodies against programmed cell death-1 (PD-1), avelumab, an antibody against the ligand of PD-1, PD-L1, and the CTLA-4 antibody ipilimumab. All these antibodies are approved by US Food and Drug Administration for treatment of different cancers [245]. Clinical trials with pembrolizumab in MCC patients showed 56% objective response rate (ORR) and the ORR in VP-MCC patients (*n* = 16) and VN-MCC patients (*n* = 9) was 62% and 44% respectively and 59% (*n* = 32) and 53% (*n* = 18), respectively [246,247]. The ORR to nivolumab was also regardless of the viral state of the tumor [248]. The ORR in VP-MCC patients (*n* = 23) and VN-MCC patients was 26% and 46%, respectively of the VN-MCC patients (*n* = 13), while in another study including 46 VP-MCC and 31 VN-MCC patients, the ORR was 28% and 36%, respectively [249]. Treatment of five MCC patients with ipilimumab indicated a positive activity of ipilimumab, but the viral state of the patients was not reported [250]. In conclusion, the response of MCC patients to checkpoint inhibitors seems to be independent of the viral state of the tumor, urging the development of VP-MCC specific therapy.

### 7.1. Vaccines

Serological studies in different countries demonstrated that most of the adult population have antibodies against MCPyV VP1 and infection occurs early in childhood (see Section 1.2). Although prophylactic vaccination with purified VP1 or virus-like particles at early age can prevent viral infection and later on development of VP-MCC, no such vaccines exist for the moment. The reason for this is probably that although the virus establishes a life-long infection in ~80% of the people virus, only a minority will develop VP-MCC and unfortunately, this makes the development of a MCPyV vaccine not very lucrative for pharmaceutical and biotechnology companies. Another option is preventive vaccination with T antigens. Approximately 1% of non-MCC patients have low titer of antibodies against these oncoprotein probably because of low viral activity or latent infection and the fact that LT is a nuclear protein, reducing its processing and presentation by HLA class 1 [251]. However, seroprevalence and titers increase significantly in MCC patients [221]. A possible pitfall with LT vaccination is that HLA class 1 is downregulated in 84% of MCC tumors, and HLA class 1 expression was significantly lower in VP-MCC than in VN-MCC [195], suggesting reduced antigen presentation. Studies with LT/sT proteins as prophylactic vaccines are lacking, but DNA vaccines coding sT or the N-terminal domain of LT have been tried out in mice. DNA vaccination generated a specific T cell immune response in mice and potent protective and therapeutic antitumor effects in a preclinical murine MCC tumor model [252,253,254]. Therapeutic vaccination to improve the immune system of MCC patient offers another alternative. For an excellent recent review, the reader is referred to [251]. Therapeutic vaccination with MCPyV T antigens can support improperly primed T cells and stimulate naive CD T cells. However, administration of whole sT protein or tumor variants of LT may promote tumorigenesis. This can be circumvented by using peptides of these proteins or mutant forms (e.g., a non-RB binding LT) that lost their oncogenic properties.

### 7.2. CRISPR/Cas9-Based Methodology

Depletion of T antigens expression by the CRISPR/Cas9 technology was shown to impair proliferation and induce apoptosis of VP-MCC cell lines [112]. Development of a CRISPR/Cas9-based therapeutic tool against T antigens in VP-MCC is a possibility. Challenges of the method to overcome are delivery of the gRNA, efficiency, and accuracy of the mutation.

### 7.3. RNA Interference Based Treatment

shRNA-mediated knockdown of T antigens expression has been successfully used in cell culture. Intratumorally injected shRNA against viral oncoproteins either as nanoparticles or vector-based are being studied [255,256] and clinical trials are being performed (e.g., NCT01505153). (Sub)cutaneously located primary MCC tumors should be easily accessible for injections with shRNA.

### 7.4. Anti-Viral Drugs

No specific inhibitors of PyV LT or sT exist, although small molecules that inhibit the ATPase activity of SV40, BKPyV and JCPyV have been described [257,258]. Since MCC tumors express tLT devoided of the ATPase domain, these drugs are not applicable for VP-MCC. However, some molecules not specific for MCPyV show anti-viral activity in cell culture and xenograft models. The malaria drug artesunate reduces growth and survival of VP-MCC cells in vitro and VP-MCC tumors in a xenograft mouse model, but had no or little effect on primary fibroblasts, melanoma cell lines, and non-classical VN-MCC cell lines [259]. Artesunate down-regulated expression of LT, but only in one VP-MCC cell line was the sensitivity towards artesunate reduced upon knockdown of LT expression. Artesunate has entered clinical trials with solid tumors cancer patients other than MCC patients and was well tolerated and modest clinical activity was observed [260]. DDL3 could be a therapeutic target in patients with VP-MCC because DLL3 expression is higher than in VP-MCC [237]. Treatment of a 67-year-old patient with metastatic MCC who received three doses of DLL3-targeting antibody-drug conjugate rovalpituzumab tesirine (Rova-T) had partial response with 57% decreased of the target lesions [237]. It was not specified whether this patient had VP-MCC. Nuclear expression of survivin in MCC is associated with poor prognosis [261]. MCPyV LT upregulates survivin levels and expression of survivin is necessary for VP-MCC cells to survive [159]. The survivin inhibitor YM155 reduced growth of some VP-MCC xenograft tumors and was nontoxic in mice, suggesting YM155 is an attractive drug to treat VP-MCC patients [159,262]. Several clinical trials using survivin vaccination are registered (e.g., NCT00108875, NCT02851056, NCT00573495, NCT03879694), but none include MCC patients. MCPyV LT’s interaction with HSC70 is important for inactivation of RB1 [12]. MAL3-101, a specific inhibitor of DnaJ-stimulated HSC70 ATPase activity [263], induces apoptosis in some MCC cell lines and inhibits tumor growth of xenografted VP-MCC WaGa cells without toxic side effects [264]. However, MAL3-101 triggered apoptosis of MCC cells irrespective of the presence of MCPyV, but cells with high HSC70 expression levels were more sensitive. MAL3-101 appears to be a candidate to treat MCC, independent of the viral state, but to our best knowledge, no clinical trials are ongoing. VP-MCC have higher expression of ADAM10 and 17 compared to VN-MCC [174]. TIM-3 is shed by both ADAM 10 and 17 and blocking TIM-3 by antibodies reduced PD-1 expression and increased cytokine production [265]. Therefore, TIM-3 seems to dampen the immune system [266]. Moreover, ADAM 10 cleaves HLA class 1 [267]. Thus, ADAM 10 and 17 inhibitors may stimulate the immune system and could be used for the treatment of VP-MCC. VP-MCC cell lines and MCC tumors do not express STING (see Section 4; [215]). Treating MKL-1 and MS-1 cells that stably express STING responsive to the STING agonist DMXAA not only restored the induction of interferons and proinflammatory cytokines and chemokines, but stimulated PD-L1 expression, T cell migration and activation, and triggered cell death in vitro [215]. The authors suggested that introducing STING by e.g., an adenovirus-based vector in MCC together with DMXAA could be used to treat VP-MCC patients.

## 8. Conclusions and Future Perspectives

The first HPyVs were discovered in 1971 and despite their ability to transform cells and induce tumors in animals, their role in human cancer remains unclear [268,269,270,271]. It was not until 2008 when the lab of Chang and Moore isolated MCPyV that the first HPyV that can cause cancer was identified [62]. Together with raccoon polyomavirus, they are the only two PyVs known to cause cancer in their natural host [272]. Despite our increase in understanding MCPyV’s role in MCC, many important questions remain unsolved. The uncertainty about the genuine cell(s) of origin of VP-MCC has hampered studies to scrutinize the exact roles of the T antigens in tumorigenesis. Transgenic mice studies have failed to ubiquitously demonstrate that sT can cause MCC. Research questions related to the biology of this virus (route of infection, transmission, spreading, cell tropism, replication) need to be solved. Efficient cell cultures for MCPyV are lacking, although human dermal fibroblasts can sustain viral replication [44]. Another enigma is why only about 0.5–1 individuals/100,000/year develop MCC with 80% of them being VP (for recent reviews see [49,64]), although most people are infected with MCPyV (see Section 1.2) and seem to chronically shed virus from the skin [33]. MCPyV induced MCC might just be an unfortunate, non-intendent event. An animal model to study virus-induced MCC is lacking (sT transgenic mice do not develop MCC and xenograft studies are usually performed in immune deficient mice). Cases of MCC has been described in other mammals, including cat, dog and steer [273,274,275,276,277,278]. It is not known whether a polyomavirus might be involved in these MCC, but bovine and canine polyomaviruses have been described [279,280], while LiPyV, originally isolated from human skin [281], was detected in feces of cats [282]. However, the sT and LT of bovine PyV, dog PyV and LiPyV share <50% homology with the corresponding proteins of MCPyV. T antigens of gorilla and chimpanzee PyVs, which are phylogenetically more closely related [1], are 80% identical to the MCPyV T antigens and DnaJ, RB, MUR, LSD, and PP4 domains are conserved, but MCC has not been described in the apes. If MCC in any of these animals has a polyomavirus etiology, they could be used as model systems to improve our knowledge on virus-induced MCC and to test out novel therapeutic strategies. Other gaps of knowledge are related to the clinics. VP-MCC specific biomarkers that can be used in diagnosis, prognosis, and response to treatment are currently lacking so that determining the viral state of the tumor depends on detecting the presence of viral DNA, RNA or T antigens in biopsies. Specific therapy for VP-MCC does not yet exist and will require identification of potential therapeutic targets in VP-MCC. Proteomics of VN- and VP-MCC may allow identification of tumor-specific proteins that can be targets for therapy or useful biomarkers. The discovery of MCPyV as a causative agent of MCC has stirred up MCC research and the next decennia will certainly further increase our knowledge and lead to the development of improved treatment for this aggressive cancer.

## Figures and Tables

**Table 1:** Supportive and contradictory arguments for the different hypotheses on cell of origin of MCC. The suspected cell of origin of VN-MCC and VP-MCC is indicated.

Cell of Origin	Supporting Cell of Origin	Arguing Against Cell of Origin
Merkel cell (VN-MCC)	Neuroendocrine granules [80] CK20 expression [72] Piezo 2 expression [81] Other neuroendocrine markers such as CD56, chromogranin A, synaptophysin, insulinoma-associated protein 1 [82]	Epidermal location [83] Postmitotic cells [84] Diffusely arranged skeleton [82] c-KIT, PAX-5, SCF, BCL2, CD24 are commonly expressed in MCC, but absent in MC [85] Neural cell adhesion molecule L1 (CD171) and neurofilament in MCC but not MC [86] Vasoactive intestinal peptide and metenkephalin expressed in MC but not MCC [87] Diffuse CK20 staining in MC; dot-like staining in MCC [88] CK20-positive MC not infected by MCPyV [82] Mouse models of VN- and VP-MCC using MC-specific Cre drivers do not develop MCC [89]
(Epi)dermal stem cell (VN-MCC)	Neuronal cell markers [82] CK14 expression [90] CK19 expression [91,92] SOX-2 expression [93] Mitotic potential [85] Other epidermal markers such as EMA, CK56, and EpCAM [87] VN-MCC harbor UV mutational signature characteristic of epidermal-derived cancers [77]	Expression of B-cell markers [85] SOX-2 more widespread expression [82,85] Absence of MCPyV DNA in these cells [85] CK19 also found in MC [94]
Pro/pre-B cell (VP-MCC)	B-cell specific lineage factors such as PAX-5, c-KIT, TdT *, SCF, RAG1 [85] Expression Ig in VP-MCC [95] IgH and Igκ rearrangements in VP-MCC [96] MCC regression with idelalisib treatment [97]	Neurendocrine granules [80] Location of MCPyV transduction [85]
Skin-derived precursors (VN-MCC)	Dermal location [82] Broad differentiation	Absence of MCPyV DNA in these cells [85]
Dermal fibroblasts (VP-MCC)	Permissive for MCPyV [44] Mutational burden similar to VP-MCC [77] Mutational signature similar to VP-MCC [77] Only cell type that can be transformed by sT in vitro [98,99,100]	Gene expression profile [85] Neuroendocrine differentiation [85] Expression of B-cell markers [85] Lack of MCPyV DNA in HDF adjacent to VP-MCC [85]
Keratinocytes (VN- and VP-MCC)	Keratinocytes and MCs are derived from the same epidermal progenitor cell [101] Transgenic mice expressing LT or sT in keratinocytes can result in oncogenic effects [76] Mutational burden of VN-MCC is in line only with two other cancers, both keratinocyte-derived skin cancers [77] VN-MCC have mutations in *NOTCH1*, *HRAS* and *FAT1*, which are frequent in squamous cell carcinoma [77]	

* TdT expression in 65% of all examined MCC and expression is significantly correlated with the presence of MCPyV.; PAX-5:90% of all examined MCC.

**Table 2:** MCPyV LT and sT interaction partners and their role in the life cycle of MCPyV. See text for details.

T Antigen	Protein	Functional Class	Biological Role	Reference
sT	abhydrolyse domain containing 12 (ABHD12)	metabolism	unknown	[129]
sT	ankyrin repeat domain 13A (ANKRD13Aa)	protein stability	unknown	[129]
sT	ATPase sarcoplasmic/endoplasmic reticulum Ca^2+^ transporting 2	metabolism	unknown	[129]
LT	ATP binding cassette subfamily A Member 13 (ABCA13)	signaling	unknown	[129]
sT	ATP binding cassette subfamily D member 3 (ABCD3)	signaling	unknown	[129]
LT	ATP binding cassette subfamily D member 13 (ABCD13)	signaling	unknown	[129]
sT	aryl hydrocarbon receptor interacting protein (AIP)	transcription	unknown	[129]
LT	adaptor related protein complex 2 subunit A and M (AP2A1 and M1)	intracellular transport	unknown	[129]
sT	ADAM metallopeptidase domain 9 (ADAM9)	cytoskeleton/extracellular matrix	unknown	[129]
LT	ataxia telangiectasia mutated (ATM kinase)	DNA replication and repair	LT phosphorylation	[14]
LT, sT	BCL2 associated anthanogene 2, 3 and 5 (BAG2, 3 and 5)	protein stability/apoptosis	unknown	[129]
LT	bromodomain protein 4 (Brd4)	cell cycle/DNA replication	viral genome replication	[130,131]
sT	cadherin 1 (CDH1)	cytoskeleton/extracellular matrix	unknown	[129]
LT	casein kinase 2 beta (CK2β)	Signaling	unknown	[129]
sT	cathepsin B (CTSB)	protein stability/modification	unknown	[129]
LT	caveolae associated protein 2 (CAVIN2)	intracellular transport	unknown	[129]
sT	CCHC-type Zinc finger nucleic acid binding protein (CNBP)	transcription	unknown	[129]
sT	cell surface glycoprotein 44 (CD44)	cell-cell interaction, cell adhesion, migration	unknown	[129]
sT	cell division cycle 20 (CDC20)	cell cycle	sT-mediated phosphorylation of 4E-BP1	[129,132,133]
sT	coatomer protein complex subunit γ2	intracellular transport	unknown	[129]
sT	2′, 3′-cyclic nucleotide 3′ phosphodiesterase (CNP)	nucleotide metabolism	unknown	[129]
LT	DEAD-box helicase (DDX24)	post-transcription/translation	unknown	[129]
sT	heat shock protein 40 members A1 and B4 (DnaJA1 and B4)	chaperone	unknown	[129]
LT, sT	heat shock protein 40 member C7 (DnaJC7)	chaperone	unknown	[129]
LT	transcription factors E2F3 and 4 (E2F3 and 4)	transcription	unknown	[129]
sT	EGF containing fibulin extracellular matrix protein 2 (EFEM2)	cytoskeleton/extracellular matrix	unknown	[129]
sT	eukaryotic translation initiation factor 4E binding protein 1 (eIF-4EBP1)	translation	disregulated cap-dependent translation which promotes tumorigenesis	[99,133]
LT, sT	emerin (EMD)	cytoskeleton	unknown	[129]
LT	family with sequence similarity 71 member E2 (FAM71E2)	unknown	unknown	[129]
sT	F-box and WD repeat domain containing 7 (Fbxw7)	protein stability	tumorigenic properties of the virus (stabilization of LT and cellular proteins)	[134,135]
LT	general transcription factor IIIC subunit 1 (GTF3C1)	transcription	unknown	[129]
LT	high density lipoprotein binding protein (HDLBP)	metabolism	unknown	[129]
LT, sT	heat shock protein 70 (HSPA1 and A4)	chaperone	cell cycle progression	[129]
sT	insulin like growth factor 2 receptor (IGF2R)	signaling	unknown	[129]
LT, sT	inhibitor of nuclear factor kappa-B kinase-interacting protein (IκBIP)	signaling	unknown	[129]
LT	karyopherin subunit α2, 3 and 4 (KPNA2, 3 and 4)	intracellular transport	unknown	[129]
sT	lysyl oxidase (LOX)	metabolism	unknown	[129]
LT	microtubulin-associated protein 4 (MAP4)	cytoskeleton	unknown	[129]
sT	membrane bound O-acetyltransferase domain containing 7	metabolism/plasma membrane lipid organization	unknown	[129]
LT	mediator complex subunit 14 (MED14)	transcription	unknown	[129]
sT	matrix metalloproteinase 14	extracellular matrix	unknown	[129]
sT	myelin protein zero like 1 (MPZL1)	signaling	unknown	[129]
sT	mitochondrial carrier 2 (MTCH2)	metabolism	unknown	[129]
sT	myoferlin (MYOF)	membrane morphology	unknown	[129]
sT	NF-kappa-B essential modulator (NEMO=IKBKG)	signaling	inhibition NFκB signaling; immune evasion	[136]
sT	Notch 2 receptor (NOTCH2)	signaling	unknown	[129]
sT	nuclear receptor binding SET domain protein1 (NSD1)	transcription	unknown	[129]
LT	prolyl 4-hydroxylase subunit alpha 3 (P4HA3)	metabolism	unknown	[129]
sT	prolyl 4-hydroxylase subunit β (P4HB)	metabolism	unknown	[129]
sT	platelet-derived growth factor receptor subunit β (PDGFRβ)	signaling	unknown	[129]
LT	PGAM family member 5, mitochondrial Ser/Thr protein phosphatase (PGAM5)	signaling	unknown	[129]
sT	progesterone receptor membrane component 2 (PGRMC2)	signaling	unknown	[129]
LT	phosphatidylinositol-5-phosphate 4-kinase type 2 beta (PIP4K2β)	signaling	unknown	[129]
LT	protein phosphatase 2 scaffold subunit α (PP2AR1α)	signaling	unknown	[129]
sT	protein phosphatase 2 catalytic subunit α and β (PPP2CA and CB)	Signaling	mutation in PP2A binding site had no effect on the known activities of sT	[17,136,137]
sT	PRA1 domain family member 2 (PRAF2)	intracellular transport	unknown	[129]
sT	protein phosphatase 2 regulatory subunit Aα and Aβ (PP2R1A and B)	signaling	unknown	[138]
sT	protein phosphatase regulatory subunit 1 (PP4R1)	signaling	microtubule destabilization and cell motility (metastasis?); inhibition NFκB signaling (immune evasion?)	[17,136,139,140]
sT	protein phosphatase Mg^2+^/Mn^2+^ dependent 1A, 1B and 1G (PPM1A, B and G)	signaling	unknown	[129]
sT	proteasome 26S ATPase 2,3 and 4 (PSMC2, 3 and 4)	protein stability	unknown	[129]
LT	caveolae associated protein 1 (PTRF)	transcription	unknown	[129]
sT	pituitary tumor-transforming gene 1 protein-interacting protein (PTTPG1P)	intracellular transport	unknown	[129]
sT	Rab18 (RAB18)	signaling	unknown	[129]
LT	Retinoblastoma protein 1 (RB1)	cell cycle	cell cycle progression	[111,115,117]
sT	ribonuclease/angiogenin inhibitor 1 (RNH1)	transcription/translation	unknown	[129]
sT	ribosomal protein L21	translation	unknown	[129]
sT	ribosomal protein S27 like	translation	unknown	[129]
LT	recitulon 4 (RTN4)	intracellular transport	unknown	[129]
LT	sphingosine-1-phosphate lyase 1 (SGPL1)	metabolism	unknown	[129]
sT	secreted protein acidic and cysteine rich (SPARC)	extracellular matrix	unknown	[129]
sT	sulfide quinone oxidoreductase (SQRDL)	metabolism	unknown	[129]
LT	signal recognition particle 14 (SRP14)	intracellular transport	unknown	[129]
LT, sT	signal recognition particle receptor subunit b (SRPRB)	intracellular transport	unknown	[129]
sT	ser/thr kinase 38 (STK38)	signaling	unknown	[129]
LT, sT	STIP1 homology and U-box containing protein 1 (STUB1)	protein stability	unknown	[129]
sT	surfeit 4 (SURF4)	intracellular transport	unknown	[129]
LT	Ubiquitin-specific protease (USP7)	protein stability	inhibition viral DNA replication	[127]
LT	transcription elongation factor B subunit 1 (TCEB1)	transcription	unknown	[129]
LT	transcription factor DP1 (TFDP1)	transcription	unknown	[129]
sT	translocase of inner mitochondrial membrane 8A (TIMM8A)	intracellular transport	unknown	[129]
sT	transmembrane protein 165 (TMEM165)	protein glycosylation	unknown	[129]
sT	thioredoxin related transmembrane protein 3 (TMX3)	protein folding	unknown	[129]
sT	toll interacting protein (TOLLIP)	signaling	unknown	[129]
LT	tripartite motif containing 38 (TRIM38)	protein stability	unknown	[129]
LT	testis-specific Y-encoded-like protein 1 (TSPYL1)	transcription	unknown	[129]
sT	tubulin α1 (TUBA1B)	protein folding and gap junctions	unknown	[17]
sT	tubulin β2α (TUBB2A)	mitosis and intracellular transport	unknown	[17]
LT, sT	upregulated during skeletal muscle growth 5 (USMG5)	nucleotide synthesis	unknown	[129]
LT	VPS39 subunit Of HOPS complex (Vam6p)	intracellular transport	role in DNA replication (?)	[18,129]
LT	VAMP associated proteins A and B (VAPA and VAPB)	intracellular transport	unknown	[129]
sT	vitamin K epoxide reductase complex subunit 1 (VKORC1)	metabolism	unknown	[129]
LT	vacuolar protein sorting-associated protein 11 homolog (VSP11)	intracellular transport	unknown	[129]

LT and sT induce microRNAs that target mRNAs for proteins involved in autophagy.

## Abbreviations

ALTO	Alternate frame of the LT open reading frame
HLA-1	Major histocompatibility complex class 1
HPyV	Human polyomavirus
LSD	Large T antigen stabilization domain
LT	Large T antigen
MC	Merkel cell
MCC	Merkel cell carcinoma
MCPyV	Merkel cell polyomavirus
miR	MicroRNA
MUR	MCPyV T antigen unique region
NCCR	Non-coding control region
NLS	Nuclear localization signal
OBD	Origin binding domain
ORI	Origin of replication
ORR	Objective response rate
PD-1	Programmed death 1
PD-L1	Programmed death ligand 1
PP	Protein phosphatase
PyV	Polyomavirus
RB	Retinoblastoma protein
sT	Small t antigen
TLR	Toll-like receptor
tLT	Truncated LT
VN-MCC	Virus-negative Merkel cell carcinoma
VP-MCC	Virus-positive Merkel cell carcinoma
